# No effect of preterm birth on the risk of multiple sclerosis: a population based study

**DOI:** 10.1186/1471-2377-8-30

**Published:** 2008-08-01

**Authors:** Sreeram V Ramagopalan, William Valdar, David A Dyment, Gabriele C DeLuca, Sarah-Michelle Orton, Irene M Yee, Maria Criscuoli, George C Ebers, A Dessa Sadovnick, for the Canadian Collaborative Study Group

**Affiliations:** 1 Wellcome Trust Centre for Human Genetics, University of Oxford, Roosevelt Drive, Headington, Oxford, OX3 7BN, UK; 2 Department of Clinical Neurology, University of Oxford, Level 3, The West Wing, The John Radcliffe Hospital, Oxford, OX3 9DU, UK; 3 Department of Medical Genetics, University of British Columbia, G920, Detwiller Pavilion, VCHA – UBC Hospital, 2211 Wesbrook Mall, Vancouver, British Columbia, V6T 2B5, Canada; 4 Faculty of Medicine, Division of Neurology, University of British Columbia, G920, Detwiller Pavilion, VCHA – UBC Hospital, 2211 Wesbrook Mall, Vancouver, British Columbia, V6T 2B5, Canada

## Abstract

**Background:**

Genetic and environmental factors have important roles in multiple sclerosis (MS) susceptibility. A clear parent of origin effect has been shown in several populations, perhaps resulting from factors operating during gestation. Preterm birth (birth at less than 37 weeks gestational age) has been shown to result in long-term health problems, including impaired neurological development. Here, in a population-based cohort, we investigate whether preterm birth increases the risk to subsequently develop MS.

**Methods:**

We identified 6585 MS index cases and 2509 spousal controls with preterm birth information from the Canadian Collaborative Project on Genetic Susceptibility to MS. Rates of individuals born preterm were compared for index cases and controls.

**Results:**

There were no significant differences between cases and controls with respect to preterm births. 370 (5.6%) MS index cases and 130 (5.2%) spousal controls were born preterm, *p *= 0.41.

**Conclusion:**

Preterm birth does not appear to contribute to MS aetiology. Other factors involved in foetal and early development need to be explored to elucidate the mechanism of the increased risk conferred by the apparent maternal effect.

## Background

Multiple sclerosis (MS) is a chronic inflammatory disease of the central nervous system (CNS) characterized by myelin loss, varying degrees of axonal pathology and progressive neurological dysfunction [1]. With a prevalence of about 1/1000, MS is the most common cause of acquired neurological disability in young adults of Northern European descent [1] other than trauma. The aetiology of MS remains elusive. However, like most common, complex traits, it is clear that genetic and environmental components play important roles, both independently and interactively [2].

A parent-of-origin effect (maternal) has been repeatedly observed in MS, based on data from studies of half-siblings [3], sibships including dizygotic twins [4], a large extended Dutch pedigree [5] and avuncular pairs [6] as well as a documented timing of birth effect [7]. The biological basis of this parent-of-origin effect is as yet unknown, but may arise from environmental components, gene-environment interactions and/or epigenetic modifications.

A "preterm" birth is defined as occurring at less than 37 weeks gestational age [8]. Preterm birth has been increasing in frequency over the last few decades in Western societies, now accounting for some 7% of all births in Canada [9]. Although the cause of preterm labour has no precise aetiology, several maternal risk factors have been implicated, including smoking, stress, infection and nutritional status [8]. Additionally, preterm birth has been observed to vary seasonally [10]. With improvements in perinatal care, such as surfactant therapy and ventilator strategies, survival has steadily improved [8]. This however has been accompanied by concerns about a higher risk of long term health problems among survivors [11]. Although most organs are immature, the brain and lungs are especially susceptible to the consequences of preterm birth, leading to high rates of neurological complications including cerebral palsy, cognitive deficits and neuro-sensory impairments [11]. Preterm birth has been shown to alter brain structure with reduced total cerebral tissue volume and delayed myelination compared to term births [12].

In MS, plaque load does not correlate with axonal loss [13], the accumulation of axonal loss is associated with irreversible disability [14] and natural history data show that the progressive phase is an age-dependent degenerative process [15]. Taken together, these data suggest that a possible mechanism affecting neurodegeneration/neurodevelopment may be involved in MS pathogenesis.

Here, in a population-based cohort, we investigate whether preterm birth increases the risk to subsequently develop MS. To the best of our knowledge, this is the first study on this specific topic.

## Methods

Cases and controls were identified through the population-based, longitudinal Canadian Collaborative Project on Genetic Susceptibility to Multiple Sclerosis (CCPGSMS), the methodology of which has been previously described [16,17]. Briefly, specialist MS clinics in 11 cities across Canada use standardised, personally administered questionnaires to screen individuals with MS (MS index cases) and to collect data about themselves and their families. Each participating CCPGSMS site has obtained ethical approval from the relevant institutional review board. The entire project was reviewed and approved by the University of British Columbia. The CCPGSMS combines both genetic epidemiology and molecular genetics to investigate the aetiology of MS. A key strength of the CCPGSMS is that the MS study population is derived from 14 regional clinics that have a patient pool representative of the Canadian MS population. This minimizes ascertainment biases inherent in genetic epidemiological investigations and generates a sufficiently large sample size from which significant results from both molecular and epidemiological studies can be attained. To date over 30,000 MS patients and their families have been screened.

Specific to this study, preterm data was collected by telephone interview with mothers of MS index cases and spouse controls (the term "spouse" is used generically to refer to legal spouse, same-sex partner or common law partner). Each CCPGSMS index case and spouse control (if available) was asked if he/she had a biological mother who could provide information on the individual's early life. If a positive answer was received the biological mother of the case/control was contacted by a CCPGSMS site research nurse and administered a standardised questionnaire, details of which are described in [16]. CCPGSMS site research nurses have all been trained by personnel from the central CCPGSMS centre at the University of British Columbia. An individual was classed as being born preterm if born at under 37 weeks gestational age. It is well known that maternal recall of preterm birth is sufficiently accurate for clinical and epidemiological use [18], but the validity of this information was corroborated by further information asked for on the standardised questionnaire, including data about birth weight (less than 5 pounds), length of hospital stay (average 5 to 7 days between 1940–1965, the peak time of birth of our cases and controls), and the use of an incubator, as these factors are known to be associated with preterm birth [11,19]. If a mother could not remember exact details and/or the supporting data did not fit criteria for a preterm birth, the offspring was not classed as being born preterm.

### Statistical analyses

The Chi squared test was used to assess significance when comparing index cases and controls for the rate of preterm births. The Student's t-test was used to look for differences in average gestational age. Logistic regression (using the R statistical package) was used to assess the effect of preterm birth, gender and the interaction thereof.

## Results

Complete information from maternal informants on weeks gestation at birth as well as birth weight, duration of hospital stay, and the use of an incubator was available for 6585 index cases and 2509 spousal controls. The clinical and demographic details of the index cases and controls are shown in Table 1.

**Table 1:** Clinical and demographic details of MS index cases and controls

	**MS Index Cases**	**Spousal controls**
**n**	6585	2509
**Present mean age in years (SD)**	49.0 (9.6)	50.9 (9.2)
**Sex Ratio (f:m)**	3.1:1	0.4:1
**% Relapsing Remitting MS**	67.9	/

SD = standard deviation, (f:m) = female to male sex ratio

Three hundred and seventy (5.6%) MS index cases and 130 (5.2%) spousal controls were born preterm. These values were not significantly different; *p *= 0.41 (see Table 2). Given the marked yet expected difference between sex ratios of index cases versus spousal controls because of the female preponderance in MS [20], a sex-stratified comparison was also carried out. No significant differences (*p *= 0.67 and *p *= 0.31 respectively) in the rates of preterm birth were observed when comparing female cases (280; 5.6%) and controls (46; 6.0%) – or male cases (90; 5.6%) and controls (84; 4.8%) (Table 2). This was confirmed by logistic regression analysis, which showed an effect of sex on the risk of MS (*p *< 1 × 10^-16^) but no effect of being born preterm (*p *= 0.65), or an interaction effect between sex and preterm birth (*p *= 0.31) on the likelihood of developing the disease.

**Table 2:** Number of individuals born preterm and their average gestational age in MS index cases and controls

	**MS Index Cases**	**Spousal Controls**	***p value***
**No. of individuals born preterm (%)**	370 (5.6%)	130 (5.2%)	0.41
**No. of females born preterm (%)**	280 (5.6%)	46 (6.0%)	0.67
**No. of males born preterm (%)**	90 (5.6%)	84 (4.8%)	0.31
**Average gestational age for preterm individuals**	34.6 weeks	34.2 weeks	0.25

The average gestational age of preterm MS index cases was 34.6 weeks compared to 34.2 weeks for controls (*p *= 0.25) – see Table 2. No significant differences were also found when stratifying by sex (average gestational age female index cases born preterm = 34.7 weeks, average gestational age female controls born preterm = 34.8 weeks, *p *= 0.82; average gestational age male index cases born preterm = 34.2 weeks, average gestational age male controls born preterm = 33.9 weeks, *p *= 0.52).

There were no differences in the number of individuals born preterm between MS index cases and controls when stratifying by month of birth or when stratifying MS index cases by clinical course (*data not shown*).

## Discussion

MS is a complex neurological trait with a parent-of-origin (maternal) effect shown by several studies [3,5,6]. Intrauterine factors may potentially affect susceptibility to disease [4,7]. Preterm birth may have a seasonal basis [10] and a timing of birth effect has been observed in MS [7]. Consequences of preterm birth may be detrimental neurologically [11,12]. Taken together, it is possible that being born prematurely may affect the risk to develop MS. However, our data do not support this hypothesis.

Here, we observed no significant differences in preterm births for cases or controls, even when stratifying by sex. The mean gestational age for preterm cases and controls was also similar (Table 2). Furthermore, stratification by month of birth or clinical course found no significant differences, however this analysis may have been underpowered due to the sample size reduction upon stratification.

The clinical definition of preterm birth may not take into account detrimental aspects of foetal development associated with premature birth measured in days rather than weeks that may contribute to the onset of MS in adulthood. Development of gyri and sulci has been shown to take place well into 40 weeks of gestational age [21], and hence premature birth by even a few days may result in small changes in brain structure. However, even when analysing the data as above and including only individuals born up to and including 39 weeks gestational age, there were no differences between MS cases and spousal controls (*data not shown*). Greater understanding of foetal development and early maternal influences on the foetus and child is needed before the early origins of multiple sclerosis can be assessed to a greater extent.

This study does not rule out maternal risk factors associated with preterm birth, for example smoking and infection, from MS disease pathogenesis, as these factors may still have a role independent of any effect they may have on premature birth.

Our study may be limited in that maternal recall of data may not be as accurate as data from clinical records etc. Additionally, as we erred on the side of caution when classifying individuals as preterm, we may have missed those who were born borderline preterm and hence we may be underestimating the number of preterm births in our cohort. However, this will apply equally to cases and controls and maternal recall of preterm birth has been shown to be accurate enough for epidemiological use [18].

## Conclusion

It should be remembered that parent of origin effects can arise in a number of ways, and likely also by other means as yet undiscovered. These mechanisms include genomic imprinting [22] and microchimerism [23]. We have previously shown that the rate of microchimerism is significantly higher in affected MS twins than in unaffected co-twins [24]. As a maternal influence in disease risk does not appear to act via preterm birth, the mechanism of the increased risk conferred maternally needs to be uncovered by exploring all possible avenues.

## Abbreviations

CCPGSMS: Canadian Collaborative Study Group on the Genetic Susceptibility to Multiple Sclerosis, MS- Multiple Sclerosis

## Competing interests

The authors declare that they have no competing interests.

## Authors' contributions

GCE and ADS conceived and designed the experiments. SVR, WV, DAD, GCD, SMO, IMY, MC, GCE and ADS analyzed the data and wrote the paper. All authors read and approved the final manuscript.

## Pre-publication history

The pre-publication history for this paper can be accessed here:


